# Long-term survival outcomes of HIV infected children receiving antiretroviral therapy: an observational study from Zambia (2003–2015)

**DOI:** 10.1186/s12889-019-6444-7

**Published:** 2019-01-28

**Authors:** Jane N. Mutanga, Simon Mutembo, Amara E. Ezeamama, Xiao Song, Robert C. Fubisha, Kunda Mutesu-Kapembwa, Derrick Sialondwe, Brenda Simuchembu, Jelita Chinyonga, Philip E. Thuma, Christopher C. Whalen

**Affiliations:** 1 Department of Pediatrics and Child Health, Livingstone Central Hospital, Akapelwa Street, Livingstone, Zambia; 2 Department of Epidemiology and Biostatistics, College of Public Health, University of Georgia, Athens, Georgia USA; 3 Southern Province Medical Office, Ministry of Health, Choma, Zambia; 4 Department of Psychiatry, College of Osteopathic Medicine, Michigan State University, East Lansing, MI USA; 5 Macha Research Trust, Choma, Zambia; 6 Global Health Institute, College of Public Health, University of Georgia, Athens, Georgia USA

**Keywords:** HIV, Pediatrics, Therapeutics, Treatment outcome, Survival

## Abstract

**Background:**

In 2017, 64% of children living with HIV in Zambia accessed Antiretroviral Therapy (ART). Despite expanded ART coverage, there is paucity of information on effectiveness of pediatric ART in reducing mortality. The aim of this research is to describe treatment outcomes, measure mortality rates and assess predictors of mortality among children receiving ART.

**Methods:**

Using a retrospective cohort study design, we abstracted routinely collected clinical data from medical records of children from birth to 15 years old, who had received ART for at least 6 months at Livingstone Central Hospital in Southern Province Zambia, between January 2003 and June 2015. The primary outcome was death. Cause of death was ascertained from medical records and death certificates. Distribution of survival times according to baseline covariates were estimated using Kaplan Meier and Cox Proportional Hazards methods.

**Results:**

Overall, 1039 children were commenced on ART during the study period. The median age at treatment initiation was 3.6 years (IQR: 1.3–8.6) and 520 (50%) children were female. Of these, 71 (7%) died, 164 (16%) were lost to follow-up, 210 (20%) transferred and 594 (56%) were actively on treatment. After 4450 person years, mortality rate was 1.6/100 (95% CI: 1.4–1.8). Mortality was highest during the first 3 months of treatment (11.7/100 (95% CI: 7.6–16.3). In multivariable proportional hazards regression, the adjusted hazards of death were highest among children aged < 1 year (aHR = 3.1 (95% CI: 1.3–6.4), compared to those aged 6–15 years, WHO stage 4 (aHR =4.8 (95% CI: 2.3–10), compared to WHO stage 1 and 2. In the sensitivity analysis to address bias due to loss to follow-up, mortality increased 5 times when we assumed that all the children who were lost to follow up died within 90 days of their last visit.

**Conclusion:**

We observed low attrition due to mortality among children on ART. Loss to follow-up was high (16%). Mortality was highest during the first 3 months of treatment. Children aged less than one year and those with advanced WHO disease stage had higher mortality. We recommend effective interventions to improve retention in care and early diagnosis of HIV in children.

**Electronic supplementary material:**

The online version of this article (10.1186/s12889-019-6444-7) contains supplementary material, which is available to authorized users.

## Background

The availability of Antiretroviral Therapy (ART) for children living with HIV and implementation of universal treatment of all pregnant and breastfeeding women living with HIV (Option B+) is a game changer in the global fight against HIV [1]. In 2017, about 1.8 million children were living with HIV globally and 90% of these children lived in sub-Saharan Africa [2, 3]. Although progress has been reported in the scale-up of access to treatment for children, only 52% of children living with HIV received lifesaving ART and only 51% of HIV-exposed infants were tested for HIV by the age of 2 months as recommended by the World Health Organization (WHO) guidelines [2]. These estimates fall short of the UNAIDS 90–90-90 treatment targets, a strategy to end the global HIV epidemic. This strategy aims to achieve the following by 2020: 1) 90% of people living with HIV will know their status, 2) 90% of all diagnosed people will be on ART, 3) 90% of people on ART will be virally suppressed [4]. Although some milestones have been achieved in the provision of ART, access to early HIV diagnosis and ART among infants and children remains a challenge in high HIV burden settings [2, 5].

Similarly, the pediatric HIV program in Zambia has made tremendous progress with over 64% of children living with HIV accessing ART by the end of 2017 [6]. With a population of 17 million people and an estimated HIV prevalence of 12.9%, 72,000 were children living with HIV and 8900 were newly infected in 2017 [6, 7]. This scenario creates a large pool of children living with HIV in need of treatment. An early study done in routine care settings in Zambia demonstrated that children were diagnosed at older ages with advanced WHO stage 3 or 4 disease. The same study reported that 57% of deaths occurred within the first 90 days of treatment initiation and loss to follow-up was high [8]. Early mortality was consistently associated with lower CD4 count, younger age, low weight for height and anemia at ART initiation [8–10]. Recent studies suggest that routine care settings in resource limited settings are still confronted with critical gaps in early infant diagnosis and pediatric HIV treatment, resulting in children initiating treatment with advanced disease and at high risk of death during the first 3–6 months of treatment [11, 12]. In addition, there are concerns around interpretation of treatment outcomes in routine care settings because of the high loss to follow-up and high numbers of transfers with no mortality ascertainment [11, 13].

Although, pediatric HIV treatment programs in resource limited settings like Zambia have been treating large numbers of HIV-exposed and infected children for over 15 years, long-term survival and associated factors including barriers to long-term adherence and retention in care are not adequately elucidated and therefore the wider impact of these interventions is not clearly understood [14–16]. The aim of this research is to describe treatment outcomes, measure mortality rates and assess predictors of mortality among children receiving ART in a routine care setting over a 12-year period (2003–2015) at Livingstone Central Hospital in Southern Province, Zambia.

## Methods

Using a retrospective cohort study design, we evaluated treatment outcomes among children living with HIV who received ART between January 2003 and June 2015.

### Study setting and population

The data were collected from the Pediatric Center of Excellence clinic (PCOE) at Livingstone Central Hospital (LCH) in Southern Province, Zambia. Livingstone Central Hospital offers preventive and treatment services to nearly 1.2 million people in southern and western parts of Zambia. Pediatric HIV treatment at LCH was started in 2003 in line with national policy and in 2006 the PCOE clinic was established through a collaborative agreement between the Ministry of Health in Zambia and the Centers for Diseases Control and prevention country office (CDC) [15].

The Ministry of Health in Zambia began implementation of the WHO guidelines of treating all people who test positive for HIV in 2016. The pediatric HIV testing and treatment guidelines recommended collection of dried blood spots (DBS) cards for DNA-PCR from all HIV-exposed infants at birth, 6 weeks and 6 months of age followed by serologic testing at 9 months, 12 months, 18 months and 3 months after cessation of breastfeeding [17]. Dried blood spots cards were collected from all children who were still breastfeeding and had HIV positive serologic tests, for confirmatory testing with HIV DNA-PCR. Routine provider initiated counseling and testing was offered to guardians of all hospitalized children. Prior to 2016, pediatric HIV treatment guidelines recommended ART based on clinical and immunological criteria. First line ART regimens comprised of three antiretroviral medicines including two Nucleoside reverse transcriptase inhibitors (NRTI’s): Azido thymidine, Stavudine, Abacavir, (Tenofovir for children above 10 years old) and Lamivudine, plus one Non-Nucleoside Reverse Transcriptase Inhibitor (NNRTI), either Nevirapine or Efavirenz for ART naïve infants and children. Infants who took ART prophylaxis after delivery were commenced on Protease inhibitors (PI) based regimens (Lopinavir boosted with Ritonavir) in addition to two NRTIs. Triple NRTI based regimens were recommended for children below 3 months of age who were co-infected with Tuberculosis at baseline.

Patient data were abstracted from the medical records of children living with HIV who had received ART. The inclusion criteria were: all children aged from birth to 15 years at the time of ART initiation, children who had taken ART for 6 months or more, children who had received at least three ARVs. Children who received one antiretroviral drug for PMTCT were excluded from the study because we considered ART to be a combination of at least three antiretroviral drugs. We excluded children who started ART on or after December 31st 2014 because data collection was closed on June 30th 2015 and we included only children who took ART for at least 6 months.

### Data collection

We abstracted routinely collected data from patient medical records into a Microsoft access database. We abstracted the data starting with initial patient enrollment forms which included demographics such as date of birth, gender, date of first HIV test, mode of delivery and date when they started to take ART. We assigned unique study identification numbers to each patient record. Data abstraction was done independently by two data entry clerks and their entries were verified at random intervals by a supervisor.

### Study outcomes

The main outcome was death. We ascertained death from hospital medical records. We actively searched for death certificates and other documentation on the details of death in medical records. In some cases, details of death were verified by verbal reports from the child’s adult family members or care givers.

The secondary outcomes were: 1) Lost to follow-up, and 2) Transferred to another facility. Lost to follow-up was defined as no clinical contact for more than 3 months after they missed their last scheduled appointment. Patients who missed appointments for more than 90 days were visited by a clinic nurse and their status established. All the children who were lost to follow-up or transferred were censored on the day of their last visit to the clinic. Children who survived to the end of the study were censored on June 30th, 2015. Adolescents were censored on the date they transitioned into the adolescent clinic and were no longer seen in the pediatric clinic at 15 years of age.

### Exposure

The main exposure was initiation of ART. Since this was a retrospective cohort study, the decision to initiate ART was made by the treating physician and the child’s caregivers and not influenced by the study investigators. Covariates that were abstracted included: ART start date, the initial drug regimen and baseline laboratory measurements which included hemoglobin, CD4+ count or CD4+ percentage. Additionally, WHO clinical stage and anthropometric measurements were abstracted.

### Statistical analysis

Baseline demographics and clinical features were described by estimating medians and interquartile ranges for continuous variables and frequencies and proportions for categorical variables. Observed survival was defined as the duration of time in years from the date of ART initiation to the date of death or censoring. We estimated the distribution of survival times for the baseline covariates affecting survival using the Kaplan-Meier (KM) method. The covariates were selected apriori from literature supported by a directed acyclic model (Additional file 1). Log-rank tests were used to compare the survival curves. Cox Proportional Hazards models were used to estimate the hazard ratios for death and their 95% confidence intervals to quantify the associations. We evaluated the proportional hazards assumption using log-log plots and plots of Schoenfeld’s residuals and no violations of the assumption were found. To calculate the mortality rates, we estimated the actual person time that everyone contributed to the study during the first 6 months, 1 year, 2 years, 5 years and 10 years and constructed confidence intervals.

Multivariable Cox models were used to adjust for possible confounding. All *p*-values were two tailed. In the analysis, age was a categorical variable with 3 levels. The age categorization was based on findings from previous studies which demonstrated that infants aged less than 12 months had a different survival experience based on disease progression [5, 18]. WHO stage was a categorical variable with 4 levels in the initial analysis. In the Cox Proportional Hazards models, we combined WHO stage 1 and 2, therefore WHO stage was a categorical variable with 3 levels.

The status of children who were lost to follow-up in this study was unknown. A meta-analysis of individual patient data from HIV treatment programs in Sub-Sahara Africa found that 20% of adults lost to follow-up were actually deceased [19]. To evaluate the “worst case scenario” effect of bias due to loss to follow-up on mortality, we performed sensitivity analysis by assuming that all the children who were lost to follow-up survived for 90 days after their last visit and then died. We estimated the mortality rates for this scenario at 3 months, 6 months, 1, 5 and 10 years and compared with the main study results.

Data analysis was done using SAS version 9.4 (SAS Institute Inc., Cary, NC) and R statistical software version 3.4 [20]. We used the Survival package to plot the KM curves and Survminer package to visualize the KM curves [21, 22].

## Results

**Fig. 1:**
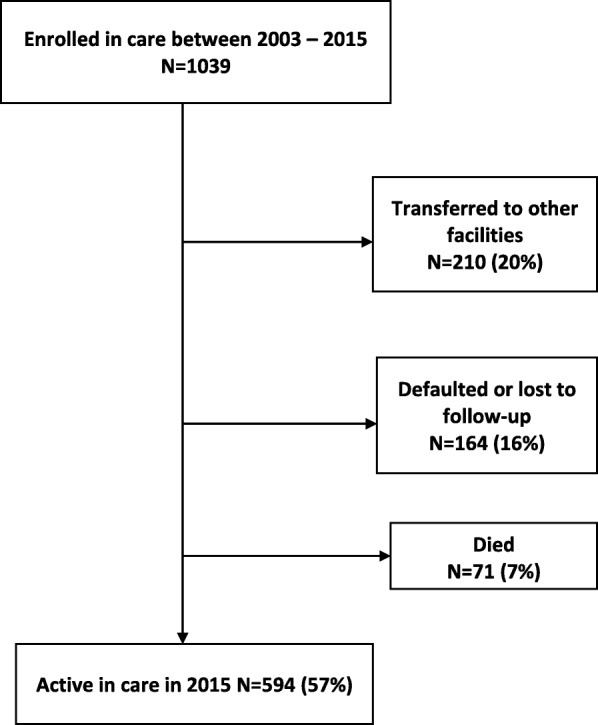
Treatment outcomes and retention in care among children on ART at LCH 2003–2015

One thousand and thirty-nine (1039) children aged less than 15 years commenced ART at LCH between January 2003 and June 2015. Overall, 71 (7%) children were confirmed to have died after commencing treatment and 594 (56%) were alive and active in care at the time of this study. A total of 164 (16%) were lost to follow-up and 210 (20%) transferred to other health care facilities (Fig. 1).

### Baseline characteristics of children started on ART

At baseline, 520 (50%) children were female and 721 (69%) were cared for by their biological mothers. A total of 179 (17%) children started treatment during their first year of life. At least 304 (29%) children were diagnosed during hospital admission and 30 (3%) were diagnosed after delivery from the delivery wards (Table 1).

**Table 1:** Baseline Characteristics of infants and Children who Received ART by Age Group - Livingstone Central Hospital, Zambia (2003–2015)

Characteristic	< 1 year	1–5 years	6–15 years	Total
	*N* = 179 (17%)	*N* = 387 (37%)	473 (46%)	*N* = 1039
Gender n(%)				
Female	84 (16%)	182 (35%)	254 (49%)	520
Male	95 (18%)	205 (40%)	219 (42%)	519
Age(years) at ART initiation Median (IQR)	0.5 (0.3–0.8)	2.0 (1.4–3.2)	10.2 (7.4–13)	3.6 (1.3–8.6)
Duration (years) on treatment Median(IQR)	2.1 (0.5–5.2)	4.2 (1.2–6.5)	4.1 (1.9–6.7)	3.8 (1.2–6.5)
Duration of time (weeks) from diagnosis to ART initiation Median (IQR)	7 (2–11)	5 (2–15)	7 (2–39)	6 (2–19)
Who is the child’s Guardian				
Mother	150 (21%)	316 (44%)	255 (35%)	721
Father	5 (11%)	8 (18%)	30 (68%)	44
Grandparent	6 (8%)	24 (32%)	46 (61%)	76
Sibling	0	1 (6%)	14 (88%)	16
other relative	5 (4%)	22 (17%)	99 (79%)	126
*missing*	*13 (22%)*	*16 (28%)*	*29 (50%)*	*56*
Point of Entry into HIV care				
Out-patients departments	22 (13%)	44 (25%)	110 (63%)	176
Inpatient Wards	80 (26%)	152 (50%)	72 (24%)	304
Delivery wards	30 (100%)	0	0	30
VCT clinic(FSU)	11 (9%)	42 (33%)	72 (57%)	127
TB clinic	3 (25%)	3 (25%)	6 (50%)	12
*missing*	*58 (15%)*	*129 (33%)*	*205 (52%)*	*390*
Educational level of caregiver				
None	2 (10%)	11 (58%)	6 (52%)	19
Primary or secondary school	113 (18%)	226 (37%)	272 (44%)	612
Some college or university	11 (18%)	16 (27%)	32 (53%)	60
*missing*	*53 (15%)*	*134 (38%)*	*163 (47%)*	*348*
Does the family have a phone				
Yes	102 (14%)	243 (35%)	358 (51%)	705
Has HIV status been disclosed to the child				
Yes	0	0	191 (100%)	191

The median age at baseline was 3.6 years (IQR: 1.3–8.6). The immunological criteria that was used to initiate treatment for children older than 5 years was CD4 count and the baseline median CD4 count was 505 (IQR: 243–948) while CD4 percentage was used for children younger than 5 years. Children aged less than 1 year had median CD4 percentage of 19.4 (IQR: 12.4–25.6) and children aged between 1 and 5 years had median CD4 percentage of 16.7 (IQR: 11.3–21.5). At least 1002 (97%) took Cotrimoxazole at baseline and 301 (30%) had a diagnosis of clinical Tuberculosis (TB) at ART initiation. Overall, 472 (46%) were WHO stage 3 and 177 (17%) were advanced WHO stage 4 (Table 2).

**Table 2:** Baseline laboratory and clinical features of infants and children who received ART at Livingstone Central Hospital, Zambia (2003–2015)

Characteristics	< 1 year	1–5 years	6–15 years	Total
	*N* = 179 (17%)	*N* = 387 (37%)	*N* = 473 (46%)	*N* = 1039
CD4 count at enrollment Median (IQR)	1028 (535–1498)	777 (444–1135)	278 (118–487)	505 (243–948)
CD4 percent at enrollment Median(IQR)	19.4 (12.4–25.6)	16.7 (11.3–21.5)	14.0 (8.3–21.2)	16.2 (10–23)
Hemoglobin at enrollment (Median IQR))	8.8 (7.8–9.7)	9.3 (8.0–10.5)	10.3 (9–11.5)	9.6 (8.3–10.9)
Taking Cotrimoxazole at enrollment				
Yes	169 (17%)	378 (38%)	451 (45%)	1000
No	9 (23%)	8 (21%)	22 (56%)	39
Drug regimen at ART initiation N(%)				
3 NRTI’s	30 (38%)	46 (59%)	2 (3%)	78
2NRTIs +1NNRTI	130 (14%)	320 (35%)	457 (50%)	907
LPV/r based	19 (35%)	21 (39%)	14 (26%)	54
Year of ART start N(%)				
2003–2005	3 (4%)	17 (24%)	51 (72%)	71
2006–2009	88 (17%)	202 (39%)	222 (43%)	512
2010–2012	62 (20%)	117 (38%)	126 (41%)	305
2013–2016	26 (17%)	51 (34%)	74 (49%)	151
Mom took ARVs for PMTCT during pregnancy				
Yes	69 (45%)	71 (46%)	14 (9%)	154
No	110 (12%)	316 (36%)	459 (52%)	885
Child took ARV prophylaxis after birth N(%)				
Yes	55 (43%)	58 (47%)	8 (10%)	123
No	124 (14%)	329 (36%)	463 (51%)	810
Nutritional status at enrollment				
Weight for height score < -3SD	36 (20%)	88 (48%)	58 (32%)	182
weight for height score > −3 SD	143 (17%)	299 (35%)	415 (48%)	857
Baseline clinical staging (WHO stage) N(%)				
WHO stage 1	52 (24%)	64 (29%)	103 (47%)	219
WHO stage 2	20 (13%)	39 (25%)	99 (63%)	158
WHO stage 3	65 (14%)	200 (43%)	206 (44%)	471
WHO stage 4	41 (23%)	78 (44%)	58 (33%)	177
missing	1	6	7	14
Diseases at baseline N(%)				
TB	33 (10%)	105 (33%)	178 (56%)	318
pneumonia	35 (21%)	68 (41%)	61 (27%)	165
Diarrhea	45 (18%)	117 (47%)	84 (34%)	247
Gestation age at birth N(%)				
Premature	4 (25%)	8 (50%)	4 (25%)	16
term	107 (22%)	201 (41%)	175 (36%)	485
unknown	68 (13%)	178 (33%)	294 (54%)	544
Mode of delivery N(%)				
C/Section	5 (28%)	3 (17%)	10 (56%)	18
SVD	105 (21%)	206 (42%)	180 (37%)	491
unknown	69 (13%)	178 (34%)	283 (53%)	530

The first line regimen for 907 (87%) children was two NRTIs and one NNRTI, and 54 (5%) were commenced on two NRTIs and a Protease Inhibitor (Lopinavir boosted with Ritonavir) while 78 (8%) children took triple NRTIs at baseline, (Azido thymidine or Stavudine, Lamivudine and Abacavir) (Table 2).

### Mortality trends

**Fig. 2:**
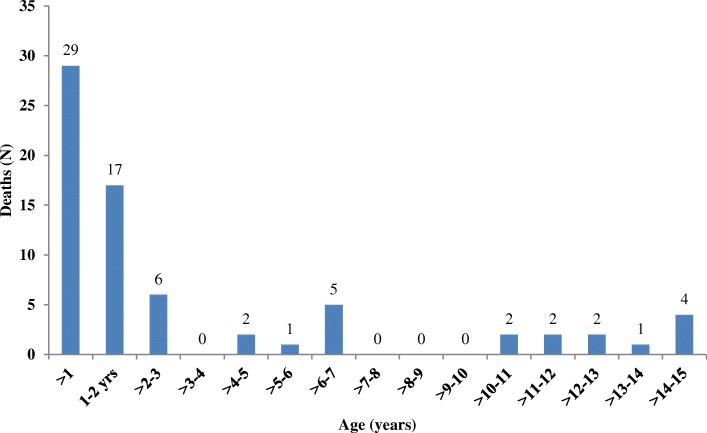
Age at time of Death Among 71 Deceased Children on ART: Livingstone Central Hospital, Zambia (2003–2015)

A total of 71 deaths were documented during the study period. The highest proportion of deaths occurred between 2007 and 2011 (Additional file 2). The largest number of deaths were among young children aged less than 12 months (*n* = 29; 41%) (Fig. 2). Of the 71 deceased children, 44% (*n* = 31) were WHO stage 4, 41% (n = 29) were WHO stage 3, 8% (*n* = 6) were WHO stage 2 and 7% (*n* = 5) were WHO stage 1. We verified causes of death from 27 (38%) death certificates. Of the verified deaths 14% (*n* = 10) were due to diarrhea, 11% (*n* = 8) severe pneumonia and protein energy malnutrition respectively and 7% (n = 5) were due to TB (Table 3).

**Table 3:** Cause of death as recorded on death certificate

Cause of death	Total number
Diarrhea	10
Severe Pneumonia	8
Severe wasting - Protein Energy Malnutrition	8
Pulmonary Tuberculosis	5
TB Meningitis	2
Gastrointestinal Bleeding	2
Malaria	2
Meningitis/encephalitis	2

We found death certificates of 27 patients. The cause of death as recorded on the death certificate is listed in the table above

Some patients had more than one cause of death listed on the death certificate

**Fig. 3:**
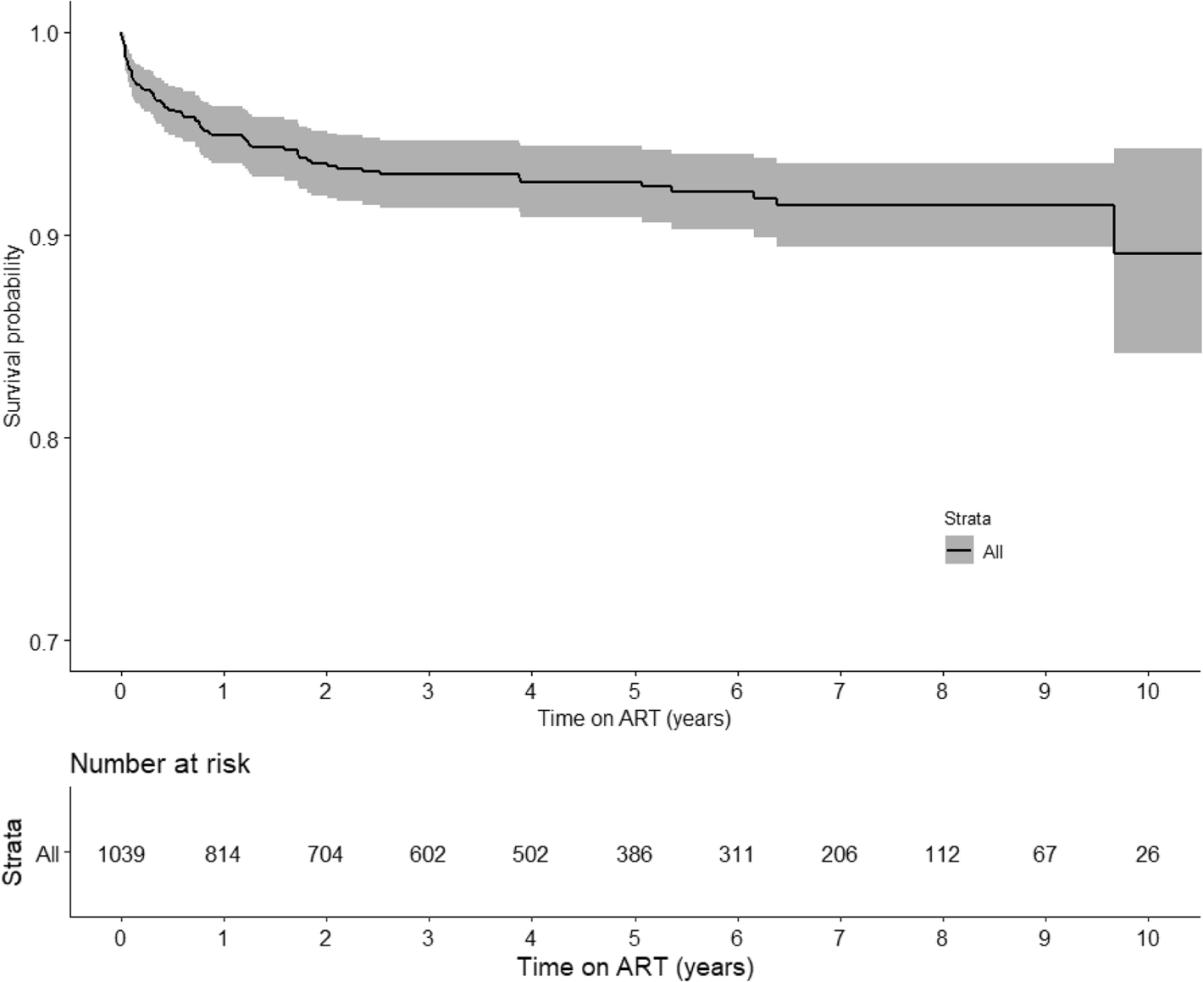
KM Survival plot of time to death after ART initiation: Livingstone Central Hospital, Zambia (2003–2015)

For an overall person-year of observation (PYO) of 4450 PY the mortality rate was 1.6/100 PYO (95% CI:1.4–1.8). Mortality was highest within the first 3 months of treatment initiation with estimated mortality rate of 11.9/100 PYO (95% CI:7.6–16.3), which accounted for 41% (n = 29) of the deaths (Table 4). The 3-month survival probability was 0.97 (Fig. 3). After 10 years of treatment, the observed mortality rate was 1.6/100 PYO (95% CI: (1.4–1.8).

**Table 4:** Mortality among children receiving ART at Livingstone Central Hospital, (2003–2015)

Duration of cART	Number of children left	Deaths	Follow-up years	Mortality Rate, deaths per 100 person-years (95% CI)
3 months	927	29	243	11.9 (7.6–16.3)
6 months	868	41	469	8.7 (6.0–11.41)
1 year	813	49	894	5.5 (3.9–7.1)
2 years	702	60	1646	3.6 (2.7–4.5)
5 years	385	66	3293	2.0 (1.5–2.5)
10 years	25	71	4450	1.6 (1.4–1.8)

In a sensitivity analyses that assumed the “worst case scenario” that all children who were lost to follow-up died 90 days after their last clinic visit, the mortality rate after 3 months of treatment was 14.5/100 person years (95% CI: 9.7–19.2). After 10 years of treatment, the mortality rate was 5.4/100 person years (95% CI: 4.7–6.1) which is 5 times higher than actual observed mortality rate (Table 5).

**Table 5:** Sensitivity analysis - effect of bias due to loss to follow-up – worst case scenario

Duration of cART	Number of children left	Deaths	Follow-up years	Mortality Rate, deaths per 100 person-years (95% CI)
3 months	956	36	248	14.5 (9.7–19.2)
6 months	901	68	478	14.2 (10.8–17.6)
1 year	822	106	908	11.6 (9.4–13.9)
2 years	710	145	1672	8.7 (7.2–10.8)
5 years	387	200	3326	6.0 (5.1–6.9)
10 years	25	229	4225	5.4 (4.7–6.1))

We assumed that all the patients who were lost to follow-up survived for 90 days after their last clinical visit then died

**Fig. 4:**
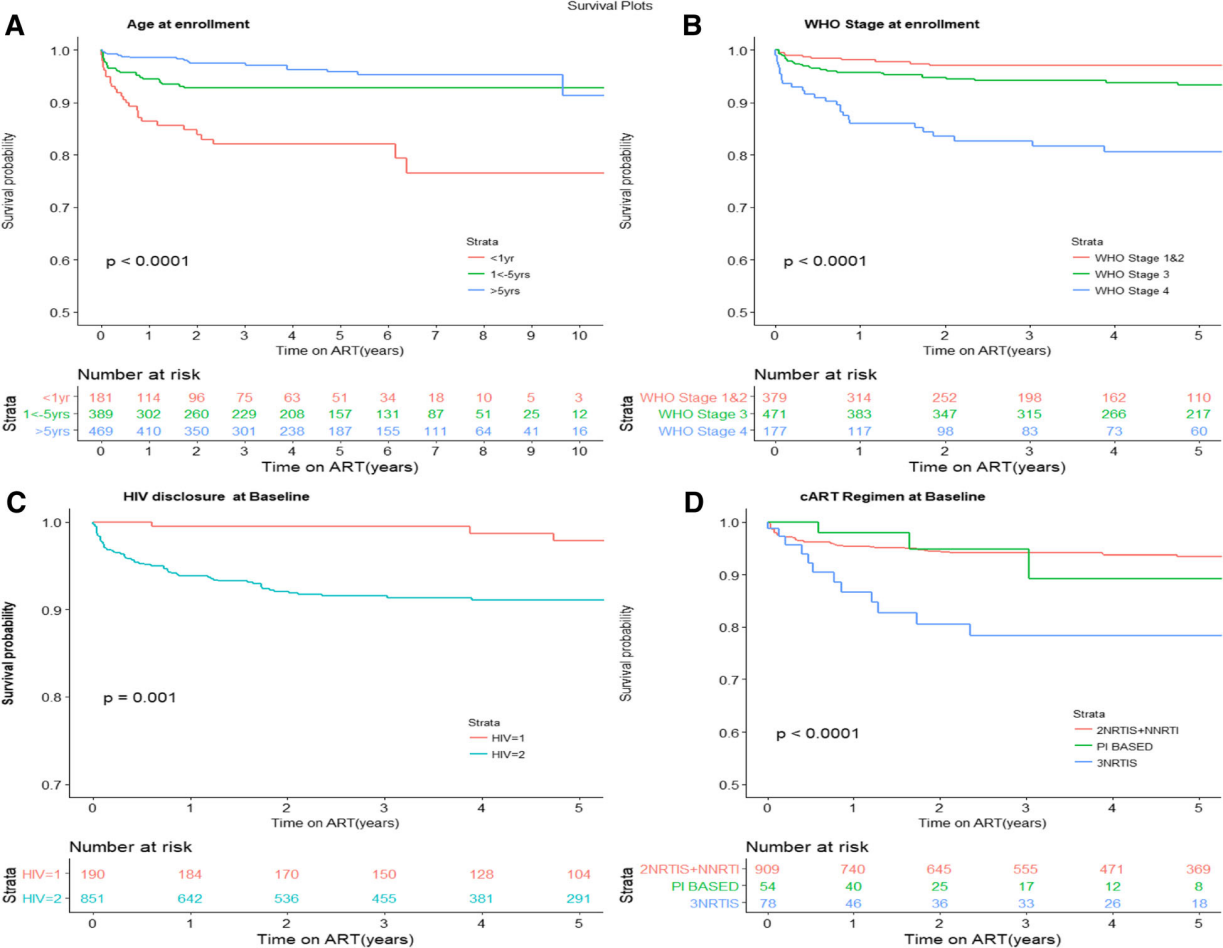
**a**-4**d**: 4A-Age at enrollment, 4**b**, WHO stage at enrollment, 4**c**, HIV disclosure at enrollment, 4**d**: ART regimen at Baseline

Children who took triple NRTI regimens had the shortest survival time (86% at 1 year) compared to children who took 2NRTI+ 1NNRTI and protease inhibitor based regimens (log rank test, *p* < 0.0001, Fig. 4 d). Other baseline factors associated with shorter survival were: children who had anemia at ART initiation (Hemoglobin < 8 g/dl) (*p* = 0.032), children of mothers who did not take antiretroviral drugs during pregnancy (*p* = 0.012), severe wasting as estimated by WAZ score < -3SD (*p* = 0.051) and infants who were given ARVs for prophylaxis (*p* = 0.056) (Additional file 3). Tuberculosis at baseline, calendar year of ART initiation and whether the infant took antiretroviral drugs for prophylaxis at enrollment were not statistically significant in the univariable Cox proportional hazards model and we did not include them in the multivariable model (Table 6).

**Table 6:** Factors Associated with Mortality Among Children on ART at Livingstone Central Hospital (2003–2015)

Predictor	N	Deaths	Unadjusted Mortality HR (95% CI)	*p*-value	Adjusted Mortality HR (95% CI)	*p*-value
Gender (N%)						
Male	519	33	Ref			
Female	520	38	1.17 (0.7–1.9)	0.5207		
Baseline age						
< 1 year	179	29	5.4 (2.9–9.9)	<.0001	3.1 (1.3–6.4)	0.0034
1-5 years	387	25	1.9 (1.03–3.5)	0.0423	1.1 (0.6–2.3)	0.7529
5–15 years	473	17	Ref		Ref	
Year of ART Initiation						
2003–2005	71	2	0.3 (0.03–2.7)	0.2778		
2006–2009	512	43	2.1 (0.8–5.9)	0.1534		
2010–2012	305	22	2.0 (0.7–5.9)	0.1948		
2013–2014	151	4	Ref			
Nutritional status at baseline						
Wasted (WAZ < =-3SD)	182	17	Ref		Ref	
WAZ > -3SD	857	54	0.59 (0.3–1.02)	0.0564	0.8 (0.6–1.5)	0.5141
CD4%						
< 15%	139	22	3.2 (0.9–10.8)	0.0561		
15–25%	285	18	2.9 (0.8–9.9)	0.08		
> =25%	225	3	Ref			
missing	390	28	3.1 (0.9–10.2)			
Baseline Hemoglobin						
< =8	705	40	1.68 (1.05–2.69)	0.0295	1.2 (0.8–2.0)	0.4025
> 8	334	31	Ref		Ref	
WHO stage at baseline						
stage 1 and 2	377	11	Ref		Ref	
stage 3	471	29	2.05 (1.01–4.06)	0.0452	1.8 (0.9–3.6)	0.1188
stage 4	177	29	6.41 (3.19–12.85)	<.0001	4.8 (2.3–10)	<.0001
missing	14	0				
TB at baseline						
Yes	318	21	0.82 (0.54–1.49)	0.6645		
No	721	50	Ref			
Regimen at baseline						
1NNRTI + 2NRTi’s	907	53	0.26 (0.14–0.46)	<.0001	Ref	
3 NRTI’s	54	4	0.37 (0.12–1.12)	0.0789	1.9 (0.9–3.7)	0.0603
LPV/R based regimen	78	14	Ref		0.9 (0.3–2.6)	0.806
Mother took ART for PMTCT						
Yes	154	17	Ref		Ref	
No	885	54	1.9 (1.2–3.4)	0.0139	1.0 (0.1–1.05)	0.0627
^a^Child know HIV status						
Yes	191	4	0.21 (0.08–0.6)	0.0029	Ref	
No	848	67	Ref		0.4 (0.1–1.05)	

^a^Applies to older children aged above 7 years’ old

Infants who started ART within the first year of life experienced shorter survival time compared to those who started treatment between 1 to 5 years and between 6 to 15 years of age (Fig. 4 a, log-rank test, *p*-value < 0.0001). The survival probability for infants aged less than 1 year was 0.93 after 3 months of treatment compared to 0.96 for those aged 1 to 5 years and 0.99 for those aged 6–15 years. In the multivariable Proportional Hazards regression, the adjusted hazards of death among children aged less than 1 year were 3.1 (95% CI: 1.3–6.4), and children aged 1–5 years aHR = 1.1 (95% CI: 0.6–2.3) when compared to the referent group 6–15 years (Table 6).

Children with severe immune-suppression (WHO stage 4) at baseline had lower survival probability of 93, 97% for the WHO stage 3 and 99% for WHO stage 1 and 2, (Fig. 4 b, log-rank test, *p*-value < 0.0001). In the multivariable Proportional Hazards regression, children with advanced WHO stage 4 had the highest hazards of mortality (aHR = 4.8 (95% CI: 2.3–10) (*p* < .0001), WHO stage 3 (aHR = 1.8 (0.9–3.6) *p* = 0.1188), compared to the referent group WHO stage 1 and 2, (Table 6).

## Discussion

In this study carried out in a routine pediatric HIV treatment setting, we found that after 12 years of treatment (2003–2015) attrition due to death was low (7%; *n* = 71). Death was highest within the first 3 months of starting ART (mortality rate: 11.7/100 PYO; 95% CI:7.6–16.3). The hazard of death was highest among children aged less than 12 months (aHR: 3.1: 95% CI: 1.3–6.4), compared to the referent group 6–15 years. Loss to follow-up was high (16%; *n* = 164).

The mortality rate observed in this study was much lower than that observed in other parts of Zambia [8, 23]. In a study conducted in an urban area in Zambia, the mortality rate was 6.6/100 PYO over 3018 PYO [8] and another study in a rural area in Zambia found a high mortality of 14.4% after 6 months of treatment [23]. These 2 studies were characterized by high mortality within the first 3 months (17.4/100 PYO) and the associated risk factors were similar to what we observed [8]. Mortality rates in our study population were also similar to a study from a rural setting in Malawi where 12 months mortality rate was 6.6/100 PYO [95% CI: 5.5–7.9] and overall mortality was 3.4/100 PYO [95% CI:2.9–4] after 5 years of follow-up [24]. Our observed mortality rate is however higher than findings from developed countries where 10-year mortality was as low as 0.3/100 PYO in the Dutch cohort [25].

The observation that hazards of death were highest within the first few months after ART initiation and then declines after the first 6 months of treatment is consistent with studies done in other parts of Zambia and in similar settings [8, 26–28]. Other studies have attributed the high early mortality to late presentation to care [29]. Studies done in routine clinic settings in resource limited settings found that the cumulative incidence of mortality during the first year of treatment among older children between 5 and 10 years of age was less than 2% to more than 45% among infants aged less than 12 months with severe disease [10]. Once children get past the initial 6 months of ART, their risk of mortality declines to very low rates especially when adherence to treatment and follow-up is optimized [11, 27, 30].

Our finding that the hazards of mortality were highest among infants and children aged less than 12 months of age is consistent with predictive models that have been done in both developed and developing countries and in fact motivated the universal ART policy by WHO to test and treat all HIV-exposed infants and children [31]. Results of a modelling study done in six countries in Sub-Sahara Africa suggested that mortality is higher among perinatally HIV-infected children than those infected through breastfeeding [32]. Perinatally HIV-infected infants have a higher risk of mortality and disease progression [5]. Early diagnosis of HIV infection and early initiation of ART improves the outcomes of infants and children [33]. Although early infant diagnosis of HIV is critical, there are still challenges in resource limited settings to diagnose children. Children who are missed by the PMTCT programs are at highest risk of mortality because they usually present to the hospital after an illness when they are already immunocompromised with high risk of mortality [34].

We assessed the causes of death in our study but only found death certificates for 27 children and no postmortem or thorough investigations had been done to ascertain cause of death. However, causes of death recorded on the few death certificates were similar to the causes of childhood deaths in Zambia, which are diarrhea, severe pneumonia and protein energy malnutrition [35]. This setting has a very high infant mortality rate (65/1000 in 2015) [36]. In the pre-ART era, AIDS related deaths led to an increase in infant mortality in high HIV burden countries. Antiretroviral therapy improved child survival and evidence is now showing that early initiation of ART leads to better clinical outcomes [33]. In a study that pooled results of clinical trial data from Zimbabwe and Uganda, the mortality risk was attributed to pre-ART risks that persist until the antiretroviral drugs reach their maximal effectiveness [27]. These early deaths have been attributed to suboptimal management of malnutrition, Tuberculosis and other related medical conditions during the early treatment stage including Immune Reconstitution Syndrome (IRIS) [11]. However, the role of IRIS in mortality during the early months of treating HIV seropositive children with ART is poorly understood [37]. Our study had insufficient evidence to support the role of IRIS as a risk factor of early mortality.

The major strength of our study is that it was done in a routine clinic setting among children commencing ART in a high HIV burden setting. This provides a real-world effectiveness of pediatric HIV treatment outcomes and provides information on important predictors of mortality. Our sample size was large and we had enough outcomes to make reasonable conclusions.

A major limitation is that there was a high proportion of loss to follow-up (16%) and many transfers (20%). This made ascertainment of mortality very challenging because some of the children who were lost to follow-up may be deceased and therefore misclassified and the mortality rates that we observed may be an underestimate. The estimated mortality rates under the “worst case scenario” assumption that all children who were lost to follow-up had died was 7 times higher after 1 year of follow-up and 5 times higher after 10 years of follow-up. A study from Malawi found that only 11% of the children who were lost to follow-up had died [13]. This study was done in an urban setting unlike our study setting which is both urban and rural. Our sensitivity analysis approach allowed us to estimate maximum mortality rates which was more informative in our setting.

Transfers are high in this clinic, which is motivated by Ministry of Health policy that encourages people to seek care at the health facility nearest to them. Studies have showed that this is an effective strategy as patients can additionally benefit from support groups and other programs that may be available at their nearest health facility [38, 39]. However, this makes it difficult to study long-term treatment outcomes because patient tracking is impossible once they transfer to another facility.

## Conclusion

We observed low attrition due to mortality among children on ART in Zambia. Loss to follow-up was high (16%) and could underestimate the mortality. Mortality was highest during the first 3 months of treatment. Children aged less than one year and children with advanced WHO disease stage had higher mortality. We recommend to program managers and clinicians to develop effective interventions to improve retention in care and strengthen early infant diagnosis of HIV. Pretreatment screening and treatment of opportunistic infections among children commencing ART needs to be strengthened to reduce early mortality.

## Additional files

Additional file 1: **Figure S1.** Directed Acyclic Graph (DAG) showing adjusted covariates of ART Initiation among Children on ART. (DOCX 108 kb)

Additional file 2: KM plots for 1) Figure S4E: Anemia, 2) Figure S4F: Mom took ART, 3) Figure S4G: Wasting (low weight at baseline, 4) Fig. 4h: Baby took NVP for Prophylaxis after delivery. (DOCX 66 kb)

Additional file 3: Trends in treatment outcomes among 1039 children on ART at Livingstone Central Hospital, Zambia: 2003–2015. (DOCX 45 kb)

## Abbreviations

AIDS: Acquired Immunodeficiency Syndrome
ART: Antiretroviral Therapy
HIV: Human Immunodeficiency Virus
LCH: Livingstone Central Hospital
NNRTI: Non-Nucleoside Reverse Transcriptase Inhibitors
NRTI: Nucleoside Reverse Transcriptase Inhibitors
PCOE: Pediatric Center of Excellence
PMTCT: Prevention of Mother to Child Transmission of HIV
WHO: World Health Organization
