# Supplementary material for: Long-term survival outcomes of HIV infected children receiving antiretroviral therapy: an observational study from Zambia (2003–2015)
Source: BMC Public Health. 2019 Jan 28;19:115. doi: 10.1186/s12889-019-6444-7 (PMC6348639; doi:10.1186/s12889-019-6444-7)
Supplement: Supplementary file 1 — Figure S1. Directed Acyclic Graph (DAG) showing adjusted covariates of ART Initiation among Children on ART. (DOCX 108 kb) [file 12889_2019_6444_MOESM1_ESM.docx]

| 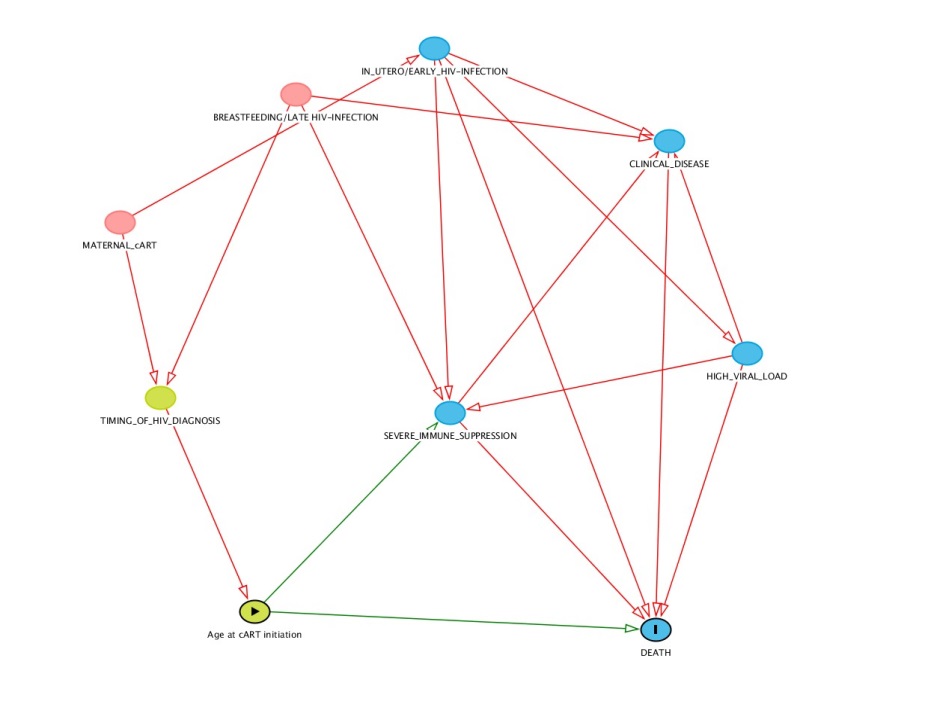 | **Legend**  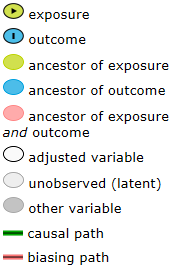 |
| --- | --- |

*After controlling for timing of HIV diagnosis, we eliminate all the biasing pathways. The major confounder of the relationship between age at cART initiation and death is timing of HIV diagnosis. All the other covariates are predictive of the outcome.
