# Supplementary figures and images for: Long-term survival outcomes of HIV infected children receiving antiretroviral therapy: an observational study from Zambia (2003–2015)
Source: BMC Public Health. 2019 Jan 28;19:115. doi: 10.1186/s12889-019-6444-7 (PMC6348639; doi:10.1186/s12889-019-6444-7)

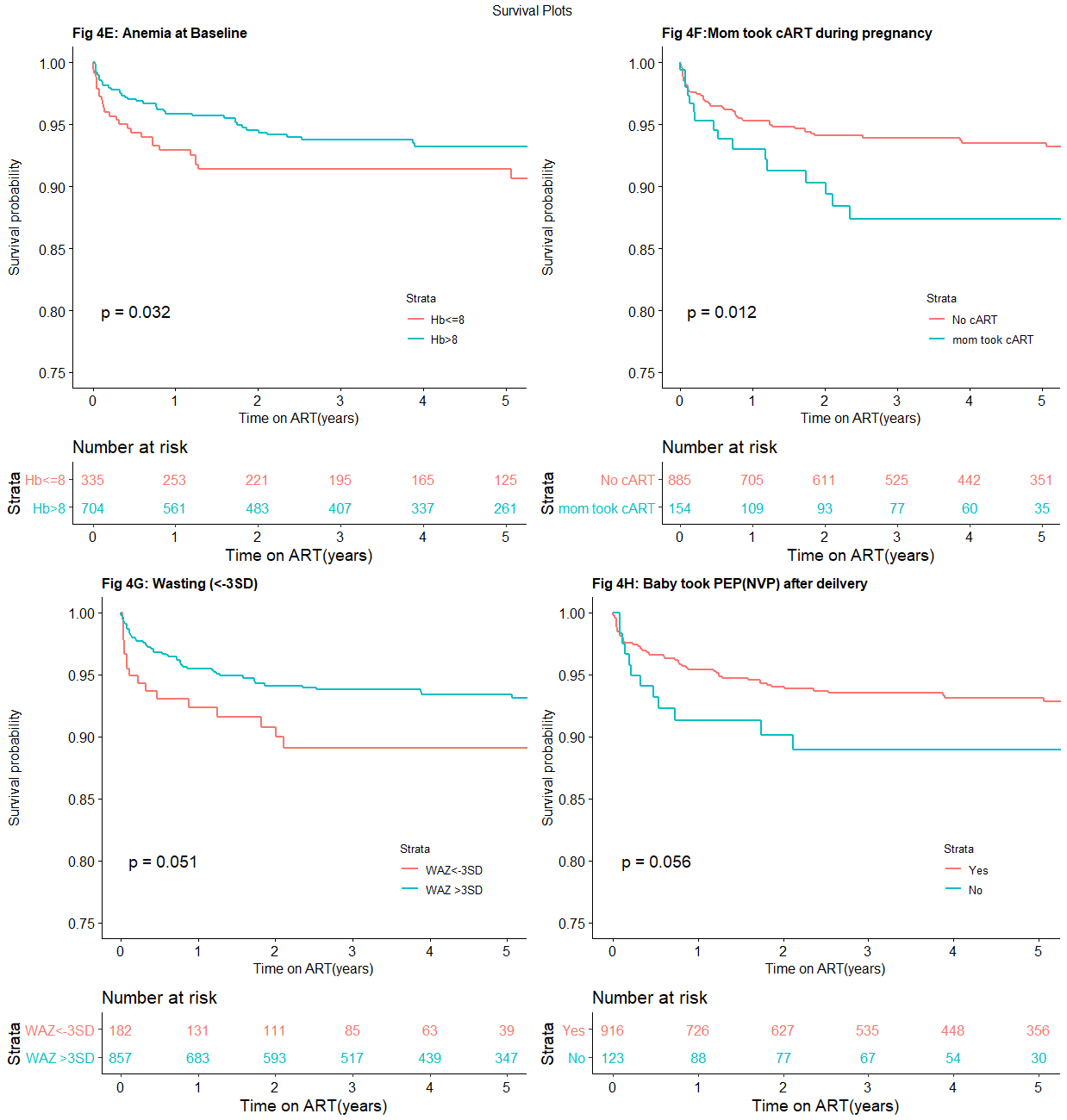

Supplement: Supplementary file 3 — Trends in treatment outcomes among 1039 children on ART at Livingstone Central Hospital, Zambia: 2003–2015. (DOCX 45 kb) [file 12889_2019_6444_MOESM3_ESM.docx]
