# Supplementary material for: Long-term survival outcomes of HIV infected children receiving antiretroviral therapy: an observational study from Zambia (2003–2015)
Source: BMC Public Health. 2019 Jan 28;19:115. doi: 10.1186/s12889-019-6444-7 (PMC6348639; doi:10.1186/s12889-019-6444-7)
Supplement: Supplementary file 3 [file 12889_2019_6444_MOESM2_ESM.docx]

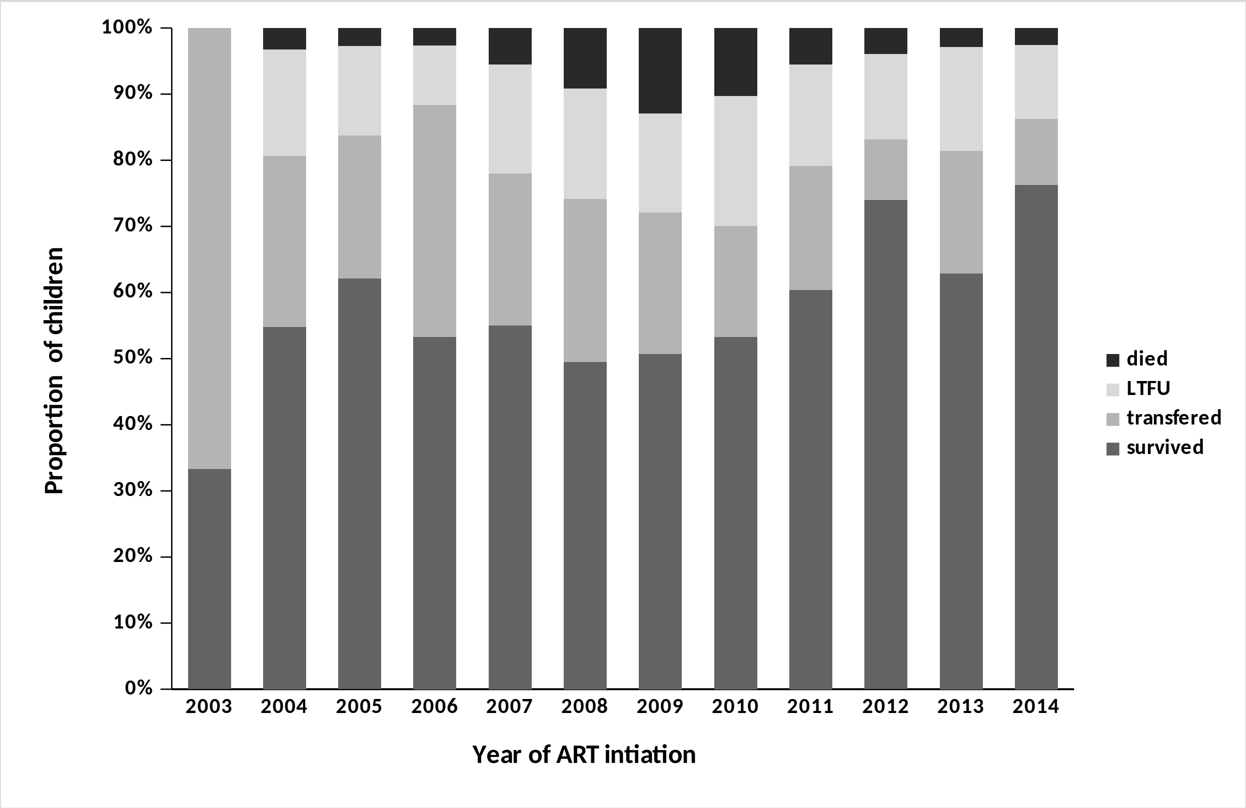

100%
90%
80%
70%
60%
50%
40%
30%
20%
10%
0%
Proportion of children
2003
2004
2005
2006
2007
2008
2009
2010
2011
2012
2013
2014
Year of ART intiation
died
LTFU
transfered
survived
